# Parasacral Transcutaneous Electrical Nerve Stimulation with Desmopressin Acetate for Treating Primary Monosymptomatic Enuresis: A Randomized Controlled Clinical Trial

**DOI:** 10.1590/S1677-5538.IBJU.2025.0093

**Published:** 2025-06-10

**Authors:** Melissa Faria Dutra, Eleonora Moreira Lima, José Murillo Bastos, Lidyanne Ilídia da Silva de Paula, José de Bessa, Amanda Lima Alves Pereira, Glaúcia Cristina Medeiros Dias, Mônica Maria de Almeida Vasconcelos, Flávia Cristina de Carvalho Mrad

**Affiliations:** 1 Universidade Federal de Minas Gerais Faculdade de Medicina Hospital das Clínicas Belo Horizonte MG Brasil Departamento de Pediatria Unidade de Nefrologia Pediátrica, Faculdade de Medicina, Hospital das Clínicas, Universidade Federal de Minas Gerais - UFMG, Belo Horizonte, MG, Brasil; 2 Universidade Federal de Juiz de Fora Departamento de Cirurgia Juiz de Fora MG Brasil Divisão de Urologia, Departamento de Cirurgia, Universidade Federal de Juiz de Fora - UFJF, Juiz de Fora, MG, Brasil; 3 Hospital e Maternidade Therezinha de Jesus da Faculdade de Ciências Médicas e Saúde de Juiz de Fora Juiz de Fora MG Brasil Hospital e Maternidade Therezinha de Jesus da Faculdade de Ciências Médicas e Saúde de Juiz de Fora - HMTJ-SUPREMA, Juiz de Fora, MG, Brasil; 4 Universidade Estadual de Feira de Santana Departamento de Urologia Feira de Santana BA Brasil Departamento de Urologia, Universidade Estadual de Feira de Santana -UEFS, Feira de Santana, BA, Brasil

**Keywords:** Enuresis, Transcutaneous Electric Nerve Stimulation, Randomized Controlled Trials as Topic

## Abstract

**Purpose:**

Approximately one-third of the children with primary monosymptomatic enuresis (PMNE) do not respond to first-line treatment. We aimed to investigate the short-term and six-month effectiveness of combining desmopressin acetate with parasacral transcutaneous electrical nerve stimulation (PTENS) in these children and adolescents.

**Materials and Methods:**

Participants aged six–17 years with PMNE were randomly assigned to receive desmopressin acetate with active or sham PTENS. Both groups participated in weekly 30-minute electrotherapy sessions for 15 weeks. The intervention group (IG) received electrotherapy at a frequency of 10 Hz and pulse width of 700 μs. A dry and wet nights calendar assessed the frequency of wet nights in the short term and six months after the intervention ended.

**Results:**

Of 66 participants, 34 were randomized to the IG. The median age was 10.3 years (8.8 – 12), and 53% were male. Intention-to-treat analysis showed a significant reduction in the frequency of wet nights after the interventions (p <0.001) in both groups, with the IG demonstrating significant improvement, immediately after the interventions (p=0.005) and after six months (p<0.001) compared to the placebo group (PG). The Kaplan-Meier survival analysis showed improvement in the IG that became more pronounced from the 15th week onwards (log-rank test, p < 0.01).

**Conclusions:**

A 15-week treatment with desmopressin acetate and PTENS significantly reduced wet nights in children and adolescents with PMNE, and this improvement was maintained six months after the interventions.

## INTRODUCTION

Primary monosymptomatic enuresis (PMNE) is defined as intermittent urinary incontinence that occurs during sleep in children five years or older. This condition is characterized by at least one episode per month lasting at least three months, with no other lower urinary tract symptoms and no more than six consecutive months of dry periods (1–3). Recent studies have identified 12 protein-coding genes, including PRDM13, S1M1, and EDNRB (4), that play a significant role in the three main mechanisms underlying PMNE: nocturnal polyuria, decreased bladder storage capacity, and an increased excitation threshold in the bladder (1–3).

Children and adolescents with PMNE may experience embarrassment, anxiety, social isolation, and a high risk of bullying as well as physical and emotional abuse. These factors can negatively impact self-esteem and quality of life (5, 6). Effective treatment is crucial. First-line treatment includes enuresis alarms and desmopressin acetate (1–3). Roughly one-third of patients may not respond to these treatments (7). Enuresis alarms have a cure rate of 40% to 70%, but many patients discontinue therapy (8). Desmopressin acetate achieves complete response in one-third of cases but has a 70% relapse rate after discontinuation (9). This highlights the need for more effective and lasting alternatives (10–12), such as parasacral transcutaneous electrical nerve stimulation (PTENS), which shows promise in treating urinary incontinence in children and adolescents (13).

PTENS is a non-invasive technique stimulating the S2 and S3 sensory nerves, potentially activating cortical areas involved in bladder control and promoting neural reconditioning via neuroplasticity (14, 15). A systematic review by Dutra et al. (11) found that PTENS, in three RCTs (16–19), reduced wet nights’ frequency in participants with PMNE. However, one RCT reported no significant benefit over controls (19). More high-quality studies are needed to confirm PTENS effectiveness for this condition.

We hypothesize that incorporating PTENS into the treatment of patients with PMNE, who are already using desmopressin acetate, could improve therapeutic outcomes and offer potential short- and long-term benefits. In this context, this study aimed to assess the short-term efficacy and six-month effectiveness of combining PTENS with desmopressin acetate for the treatment of children and adolescents with PMNE.

## MATERIALS AND METHODS

### Ethical approval

The study was registered in the Brazilian Registry of Clinical Trials (RBR—4dcjfr7) (20) and approved by the Institutional Review Board (IRB) under protocols CAAE 35990620200005149 (position statement number 4,453,428) and CAAE 35990620230015133 (position statement number 5,553,832), respectively. Legal guardians and participants aged 10 and 17 signed the Informed Consent Term and the Assent Term, respectively.

### Study Participants

One hundred eight children and adolescents aged six–17 who were diagnosed with PMNE based on the International Children's Continence Society (ICCS) criteria (1–2) were recruited. The participants were from the Multidisciplinary Outpatient Clinic for Children and Adolescents with Enuresis at the university hospitals of the two participating institutions. To be eligible, participants had to attend the clinic once a week, had a final Vancouver Symptom Score < 11 points and have not undergone treatment for enuresis for at least six months before the start of the study (21). Participants who were not included in this study or whose intervention protocols were discontinued were referred for appropriate treatment and evaluation. For socioeconomic status, participants were categorized according to the Brazilian Economic Classification Criteria (22).

Participants on medications affecting the detrusor muscle or external urethral sphincter, those with a pacemaker, polydipsia, untreated attention deficit hyperactivity disorder, severe intellectual disability, central nervous system or spinal cord injuries, and urological malformations were excluded.

### Study Design

This randomized, blinded, placebo-controlled clinical trial was conducted from June 2022 to July 2024. The study followed the Consolidated Standards of Reporting Trials (CONSORT) (23) reporting guidelines (Supplement-1).

The randomization process was conducted using Microsoft Excel's RAND function to assign participants to either the placebo group (PG) or intervention group (IG). The results were placed in sealed numbered envelopes to ensure allocation concealment and were distributed to participants during their first appointment. Participants, their families, and study researchers who assessed the interventions outcomes were blinded to participant allocation. Emergency disclosure of allocations was permitted in cases of adverse events, to ensure appropriate therapy.

A detailed medical history and physical examination were performed during the first appointment. All participants underwent renal and bladder ultrasonography, urinalysis, and urine culture.

Participants were instructed to fill out a wet and dry night calendar for 14 days to assess the frequency of enuresis. To determine nocturnal urinary output, participants wore diapers overnight for seven days. The nocturnal urine output was calculated by summing the weight of the diaper (in kg), the volume of the first urination, and the volume of any nocturia episodes. Nocturnal polyuria was defined as a nocturnal urine output that exceeds 130% of the estimated bladder capacity (EBC) (1–3), determined by averaging the largest volumes recorded over seven days (24). The EBC for children aged four–12 years was calculated as 30 × (age in years + 1), whereas it was set at 390 mL for older participants (25). Maximum voided volume was classified as small if it was less than 65% of the EBC and as large if it exceeded 150% of EBC (1).

Participants returned with the requested data completed at least 14 days after the initial appointment. Both eligible groups started the protocol by receiving desmopressin acetate with guidance on its proper use and PTENS therapy (Dualpex 961*Quark@ device). A pulsed, biphasic, and symmetrical current was used in transcutaneous application mode with the following parameters: frequency of 10 Hz, pulse duration of 700μs, maximum but comfortable current amplitude (sensory level) applied for 30 min (26), once a week (27) at the outpatient clinic for 15 weeks. In both groups, a pair of self-adhesive surface electrodes measuring 5 × 5 cm were fixed in the sacral region of S2-S3 and another in the scapular region. In the IG, only the channel positioned in the sacral region was activated, and in the PG only the scapular electrodes. These were turned on briefly for 30 s, after which the current was reduced to zero. In the IG, the pulse amplitude was adjusted if the current sensation decreased.

Participants in the PG and IG received desmopressin acetate at no cost, starting with a daily dose of 0.2 mg. Doses were adjusted according to the ICCS criteria: if participants experienced a reduction of less than 50% on wet nights, they were classified as non-responders. In such cases, the dose was increased by 0.1 mg, up to a maximum of 0.4 mg daily (1, 2). The intervention protocol was discontinued for participants who did not show improvement while receiving the maximum dosage of desmopressin acetate, which was achieved in the 7th session. It is important to highlight that desmopressin acetate should not be administered for longer than four weeks without demonstrating observable efficacy. This approach is crucial to meet our ethical responsibility to discontinue ineffective treatments and prioritize patient safety.

Compliance to desmopressin acetate was assessed by monitoring participants’ attendance at scheduled appointments related to other interventions specified in the protocol, as well as analyzing the completion of their diaries. During the reassessments, participants not only provided responses on the answer sheets but were also asked questions about any challenges they encountered regarding the use, dosage, and replacement of desmopressin acetate tablets, as well as any side effects they experienced.

Participants who showed a reduction of 50% or more in the frequency of wet nights continued in the study protocol, undergoing weekly sessions of PTENS combined with desmopressin acetate until they had completed 15 weeks.

Desmopressin acetate was gradually discontinued from the 12th session, and in the 15th session, all participants completely stopped receiving medication.

**Figure 1:**
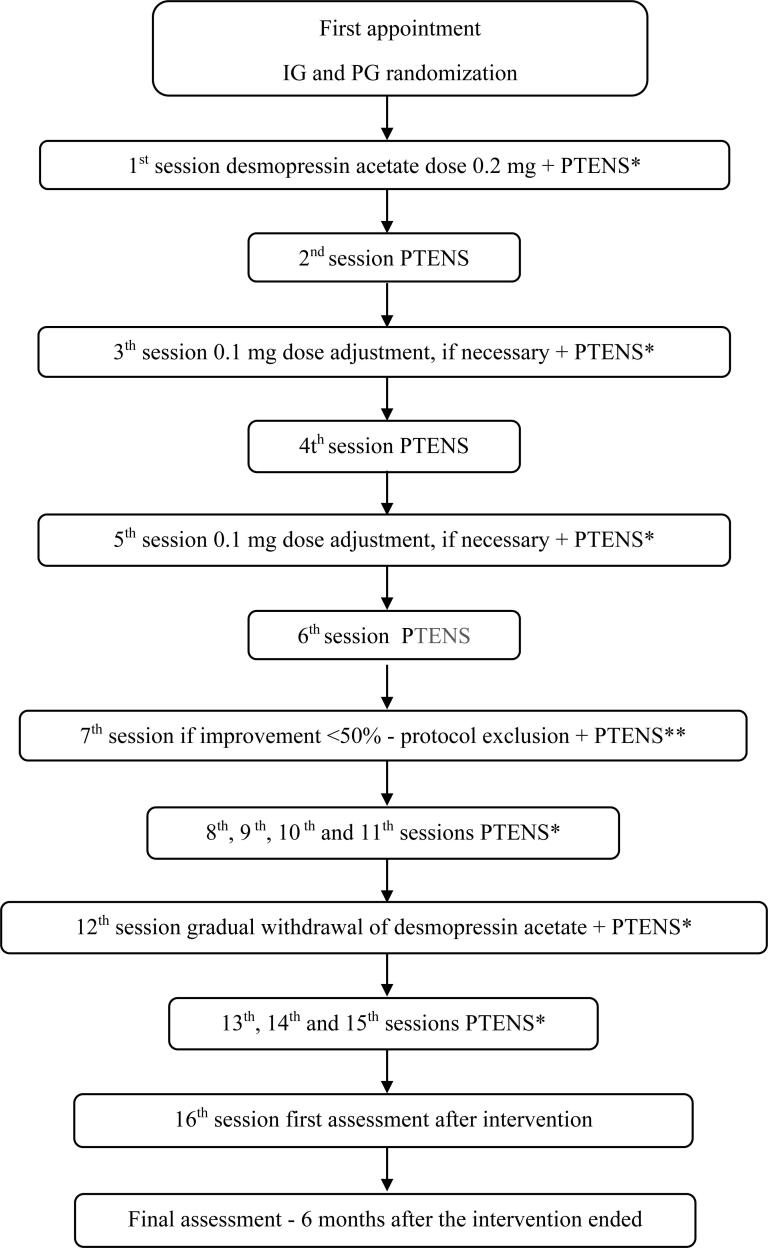
Flowchart of the intervention protocol.

The study intervention protocol is presented in Figure-1.

### Assessments

The frequency of wet nights was assessed using wet and dry night calendars over 14 days. Participants completed the questionnaire daily, beginning 14 days before treatment, throughout the 16-week interventions, and six months post-interventions.

Treatment outcomes were evaluated according to ICCS criteria (1,2), classifying improvement as no-response (<50% reduction in the frequency of wet nights), partial response (50–99% reduction in the frequency of wet nights), or complete response (100% absence of wet nights). Continued success was defined as the absence of relapse within six months after treatment completion.

Final assessments were conducted at the 16th session and again six months post-intervention. Therefore, the study protocol included a follow-up assessment six months following the completion of treatment. Throughout this period, our team closely monitored the participants, who did not receive any additional treatment.

Participants classified as no-responders after the 7th session were referred to alternative treatment options. Those still considered non-responders by the 16th session remained in the study protocol and underwent a reassessment after six months. If they continued to be categorized as non-responders after this reassessment, they were referred to other treatment options according to the ICCS-based protocol (1) of the multidisciplinary outpatient clinic.

### Sample detection power analysis

The sample detection power was calculated by comparing the outcomes of the two groups, PG and IG. In the evaluations conducted during the 16th session and six months after the interventions, we analyzed the reduction in the frequency of wet nights as a continuous variable. The percentage of improvement after the interventions was treated as a categorical variable for our calculations. As a result, the sample detection power ranged from 82.3% for the continuous variable to 78.8% for the categorical variable during the 16th session. Six months after the interventions, the sample detection power increased to 96.3% for the continuous variable and 91.3% for the categorical variable.

## Statistical Analysis

The primary analysis was performed on an intention-to-treat basis, including all participants who were randomly assigned. Data from those who discontinued treatment at the 7th week were imputed for the 16th-week and six-month post-intervention analysis.

When comparing the PG and IG, the Mann-Whitney test was used for numerical variables, and the student's t-test was used for age analysis.

Pearson's chi-square test or Fisher's exact test were used to compare categorical variables. The non-parametric Wilcoxon test was used to compare the frequency of wet nights before and after the interventions and during the six-month follow-up.

Kaplan-Meier survival analysis was used to evaluate the time until improvement occurred. The 3rd, 5th, 7th,15th was considered time units for analysis. The participants were assessed at the 16th week and at the six-month follow-up. A log-rank test was used to compare PG and IG.

For all analyses, a p-value < 0.05 was considered statistically significant. Statistical analyses were performed using IBM SPSS Statistics (version 21.0; IBM Corp, Armonk, NY, USA). G*Power version 3.1 was utilized to perform power calculations.

## RESULTS

Of the 108 participants, 66 were eligible for the study and randomly assigned to 32 in the PG and 34 in the IG. Among the 42 excluded, six had secondary enuresis, 12 had non-monosymptomatic enuresis, five had spina bifida, four had severe intellectual disabilities, one had cerebral palsy, and 14 declined to participate.

The baseline characteristics of participants are shown in Table 1. There were no significant differences in socioeconomic status between the groups. Among the participants who started the protocol, 53.1% (17/32) in the PG completed all 15 sessions, compared to 82.4% (28/34) in the IG. All participants who discontinued the protocol did so because they experienced a reduction of less than 50% in the frequency of wet nights by the seventh session; 46.9% (15/32) were from the PG. This demonstrated a significant difference compared to 17.6% (6/34) from the IG (p = 0.01).

**Table 1:** Baseline characteristics of participants and frequency of wet nights before, after interventions and at six-months follow-up.

		Total sample (n=66)	Placebo (n=32)	Intervention (n=34)	*p*- value
**Gender n (%)**					
	Male	35 (53)	16 (50)	19 (55.9)	0.63*
	Female	31 (47)	16 (50)	15 (44.1)	
**Age**					
	Mean ± SD	10.3 ± 1.9	10.4 ± 1.9	10.2 ± 1.8	0.87**
	Median (P25-P75)	10.3 (8.8 - 12)	10.3 (8.8 - 11.9)	10.3 (8.7 - 11.9)	
**Nocturnal Polyuria (NP) n (%)**					
	No	25 (37.9)	15 (46.9)	10 (29.4)	0.20***
	Yes	41 (62.1)	17 (53.1)	24 (70.6)	
**Percentage of nights with NP**					
	Mean ± SD	44.4 ± 40.7	43.7 ± 44.4	45.1 ± 37.5	0.90**
	Median (P25-P75)	42.9 (0 - 77.7)	42.9 (0 - 100)	42.9 (0 - 72.3)	
**Frequency of wet nights baseline**					
	Mean ± SD	11.3 ± 2.8	11.1 ± 3.2	11.4 ± 2.4	0.98**
	Median (P25 - P75)	12 (10 - 13)	12 (10- 13)	12 (10 - 13)	
**Frequency of wet nights after interventions**					
	Mean ± SD	6.6 ± 4.1	8.1 ± 4.3	5.3 ± 3.5	0.005**
	Median (P25 - P75)	7 (3 - 10)	9 (5 - 11)	4.5 (2 - 8)	
	*p-*value frequency comparison baseline versus after interventions	<0.001****	<0.001****	<0.001****	
**Frequency of wet nights at six-months follow-up**					
	Mean ± SD	6.0 ± 4.4	8.0 ± 4.3	4.1 ± 3.7	<0.001**
	Median (P25-P75)	6 (2 - 10)	9.5 (4.5 - 11)	3.5 (0.7 - 6.5)	
	*p-*value frequency comparison after interventions versus at six-months follow-up	0.003****	0.824****	0.008****	

P25 = Percentile 25; P75 = Percentile 75; SD = Standard Deviation; NP = nocturnal polyuri

* Chi-square test;

** Mann-Whitney test;

*** Fisher's exact test;

**** Wilcoxon test; p-value < 0.05.

Regarding the nocturnal polyuria analysis, there was no significant difference in the reduction of 50% or more in the frequency of wet nights within the groups (p = 0.23 for the PG and p = 0.11 for the IG) after the interventions. However, there was a significant difference in the proportion of participants who showed improvement between the groups. The PG showed an improvement of 41.1% (7/17), while the IG showed an improvement of 75% (18/24) (p = 0.02).

In the comparison between the beginning and after the interventions (16th week), there was a more significant reduction in the frequency of wet nights in IG (= 0.005). (Table 1) The IG showed a 52.7% reduction in the frequency of wet nights, while PG recorded a 27.6 % reduction (p = 0.006). (Table 2)

**Table 2:** Percentage improvement in the frequency of wet nights after interventions and at six-months of follow-up.

		Total sample (n=66)	Placebo (n=32)	Intervention (n=34)	*p-*value
**Reduction of the frequency of wet nights after interventions (%)**					
	Mean ± SD	40.5 ± 36.7	27.6 ± 36.9	52.7 ± 32.5	0.006**
	Median (P25 - P75)	42.9 (0 - 71.7)	21.5 (0 - 50)	59.4 (24.5 - 78.3)	
**Response after interventions n (%)**					
	No-response	34 (51.5)	22 (68.8)	12 (35.3)	
	Partial + complete response	32 (48.5)	10 (31.3)	22 (64.7)	0.007***
	Partial response	26 (39.4)	7 (21.9)	19 (55.9)	
	Complete response	6 (9.1)	3 (9.4)	3 (8.8)	
**Reduction of the frequency of wet nights at six-months of follow-up (%)**					
	Mean ± SD	44.6 ± 41.2	26.4 ± 39.2	61.6 ± 35.8	0.001**
	Median (P25 - P75)	45.5 (0 - 83.3)	20.6 (0 - 55.4)	65.5 (25 - 64.6)	
**Response at six-months of follow-up n (%)**					
	No-response	34 (51.5)	23 (71.9)	11 (32.4)	0.001***
	Partial + complete response	32 (48.5)	9 (28.1)	23 (67.6)	
	Partial response	21 (31.8)	6 (18.8)	15 (44.1)	
	Complete response	11 (16.7)	3 (9.4)	8 (23.5)	
*p-*value comparison after intervention versus at six-months of follow-up		0.08*	0.999*	0.019*	

P 25 = Percentile 25; P75 = Percentile 75; SD = Standard Deviation

* Wilcoxon test;

** Mann-Whitney test;

*** Fisher's exact test; *p*-value <0.05

At the six-month follow-up, the IG showed a significant reduction in the frequency of wet nights compared to the PG (p=0.001). (Table 1). The IG experienced a decrease in the frequency of wet nights to 61.6%, while the PG demonstrated a reduction of 26.4% (p=0.001) (Table-2).

**Figure 2:**
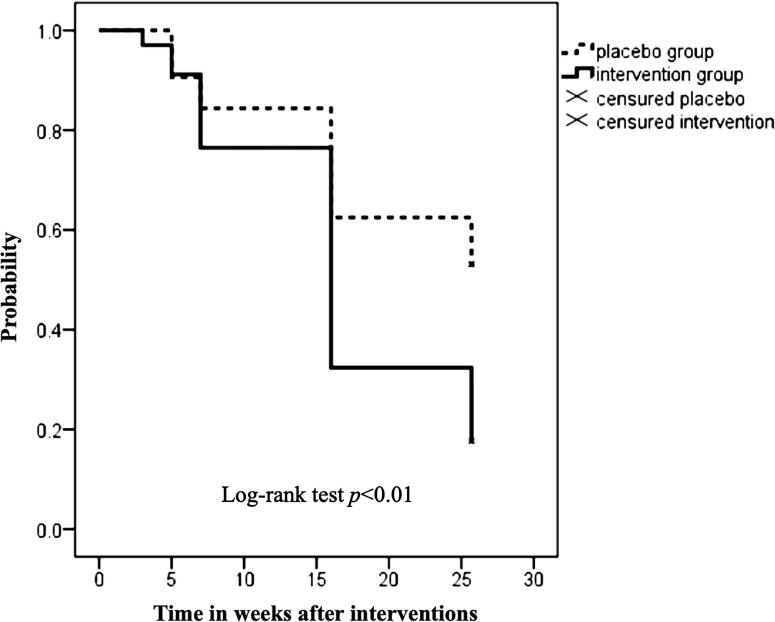
Kaplan-Meier survival analysis estimates the weeks until the frequency of wet nights decreases in the six months following the interventions

Kaplan-Meier analysis indicated that the frequency of wet nights improved earlier in the IG than in the PG, with a significant difference observed after the 15th week (log-rank test p< 0.01) (Figure-2).

In this randomized controlled trial, no adverse side effects were observed.

## DISCUSSION

Approximately one-third of patients with PMNE do not respond to first-line treatment (7). Therefore, this study aimed to investigate combination therapy and its potential benefits in managing a common condition in the pediatric population. To our knowledge, this is the first study to combine desmopressin acetate and PTENS under these conditions. After 15 weeks of combined treatment, participants in the IG exhibited a significant decrease in the frequency of wet nights, with an even more significant improvement observed six months after the intervention ended.

In our study, both PG and IG significantly reduced the frequency of wet nights (p < 0.001) after the intervention. Similar to our research, two RCTs used PTENS to treat children and adolescents with PMNE. These trials combined PTENS with standard urotherapy and demonstrated a reduction in the frequency of wet nights after interventions, with a statistically significant difference between PG and IG (16, 18). In one of these studies, the IG underwent ten sessions of PTENS treatment, resulting in a reduction of 61.8% compared to 37.3% in the PG (16). The other study using PTENS with 20 sessions, three times a week, showed a progressive decrease in the frequency of wet nights for the IG over 90 days of follow-up (18).

Nonetheless, at the six-month follow-up after the intervention ended, our study found that the IG experienced a 65.5% reduction in the frequency of wet nights. In comparison, the PG showed a 20.6% reduction. This difference was statistically significant (p= 0.001). Kaplan-Meier survival analysis revealed that the IG experienced improvement sooner than the PG. This difference was more significant between the 15th week and the six-month follow-up, with a statistically significant variance observed (log-rank test, p < 0.01). In a study by Abdelhalim and Ibrahim (17), PTENS and transcutaneous interferential electrical stimulation were compared in participants with PMNE. Both treatments significantly reduced the frequency of wet nights, but transcutaneous interferential electrical stimulation provided longer-lasting short-term results than PTENS. Lordelo et al. (26) and Veiga et al. (28) demonstrated the effectiveness of PTENS in long-term follow-up in children with overactive bladder and with non-monosymptomatic enuresis respectively. Borch et al. (29) found no immediate effect on urodynamic parameters during the use of PTENS. However, after four weeks of treatment with PTENS at home, 61% of the participants showed a significant improvement in urinary incontinence. These results suggest that PTENS can affect urinary control by neuromodulation of brain areas. A study by Netto et al. indicated that PTENS significantly impacts the central nervous system. Magnetic resonance imaging confirmed increased connectivity between the anterior cingulate and dorsolateral prefrontal cortex, possibly influencing autonomic balance for bladder control. Thus, when combined with desmopressin, the modalities would have a complementary effect (30).

The frequency of PTENS sessions was a critical factor in our study. Currently, we are in the process of defining the most effective treatment protocol. Our study used a once-a-week session frequency based on the study's findings by De Paula et al. (27). In this randomized controlled trial, children with overactive bladder and enuresis showed significant improvement in urinary urgency and enuresis in the evaluation conducted 60 days after the end of treatment (p=0.03). These data suggest that weekly sessions of PTENS may be sufficient to achieve the desired results (27). This is an important point given the difficulty of finding time for physiotherapy sessions in parents’ busy routines. Similarly, Veiga et al. (28) conducted a study in children with overactive bladder, comparing the effects of PTENS treatment administered twice a week in one group and three times a week in another group. The results showed no significant differences in symptom improvement between the two groups (28).

Individualizing PMNE treatment may improve overall efficacy and outcomes, considering its multifaceted characteristics (31). This study demonstrated a new therapeutic option for those who do not respond to desmopressin acetate alone. Results show that combining desmopressin with weekly sessions PTENS significantly reduced wet nights both in the short term and six months after the end of interventions, showing a sustained therapeutic response compared to the PG.

Despite promising results, this study has limitations. Future research should use neuroimaging and neuropsychological assessments to clarify PTENS-related neural changes. Optimizing PTENS parameters (frequency, duration, intensity) and evaluating long-term effects, including maintenance sessions, could further refine treatment strategies.

## CONCLUSIONS

After 15 weeks of treatment, a combination of desmopressin and weekly PTENS reduced the frequency of wet nights in children and adolescents with PMNE. This improvement was sustained for six months following the completion of the interventions.

## Data Availability

The data supporting the findings of this study are available from the corresponding author. Data will be made available upon request.

## ABBREVIATIONS

CONSORT: = Consolidated Standards of Reporting Trials
ICCS: = International Children's Continence Society
IG: = Intervention group
PG: = Placebo group
PMNE: = Primary monosymptomatic enuresis
PTENS: = Parasacral transcutaneous electrical nerve stimulation
RCTs: -Randomized and controlled clinical trials

## APPENDIX

**Supplement 1:** Consolidated Standards of Reporting Trials (CONSORT) checklist (23)

Section/Topic			Item No	Checklist item	Reported on page No
Title and abstract					
			1a	Identification as a randomised trial in the title	1
			1b	Structured summary of trial design, methods, results, and conclusions	1
Introduction					
Background and objectives			2a	Scientific background and explanation of rationale	2
			2b	Specific objectives or hypotheses	2
Methods					
Trial design			3a	Description of trial design (such as parallel, factorial) including allocation ratio	3
			3b	Important changes to methods after trial commencement (such as eligibility criteria), with reasons	Not applicable
Participants			4a	Eligibility criteria for participants	2,3
			4b	Settings and locations where the data were collected	2,3
Interventions			5	The interventions for each group with sufficient details to allow replication, including how and when they were actually administered	3,4
Outcomes			6a	Completely defined pre-specified primary and secondary outcome measures, including how and when they were assessed	4
			6b	Any changes to trial outcomes after the trial commenced, with reasons	Not applicable
Sample size			7a	How sample size was determined	5
			7b	When applicable, explanation of any interim analyses and stopping guidelines	Not applicable
Randomisation:					
		Sequence generation	8a	Method used to generate the random allocation sequence	3
			8b	Type of randomisation; details of any restriction (such as blocking and block size)	3
		Allocation concealment mechanism	9	Mechanism used to implement the random allocation sequence (such as sequentially numbered containers), describing any steps taken to conceal the sequence until interventions were assigned	3
	Implementation		10	Who generated the random allocation sequence, who enrolled participants, and who assigned participants to interventions	3
Blinding			11a	If done, who was blinded after assignment to interventions (for example, participants, care providers, those assessing outcomes) and how	3
			11b	If relevant, description of the similarity of interventions	3,4
Statistical methods			12a	Statistical methods used to compare groups for primary and secondary outcomes	5
			12b	Methods for additional analyses, such as subgroup analyses and adjusted analyses	Not applicable
Results					
Participant flow (a diagram is strongly recommended)			13a	For each group, the numbers of participants who were randomly assigned, received intended treatment, and were analysed for the primary outcome	Figure 1
			13b	For each group, losses and exclusions after randomisation, together with reasons	Figure 1
Recruitment			14a	Dates defining the periods of recruitment and follow-up	5
			14b	Why the trial ended or was stopped	5
Baseline data			15	A table showing baseline demographic and clinical characteristics for each group	Table 1
Numbers analysed			16	For each group, number of participants (denominator) included in each analysis and whether the analysis was by original assigned groups	6
Outcomes and estimation			17a	For each primary and secondary outcome, results for each group, and the estimated effect size and its precision (such as 95% confidence interval)	Table 1,2 Figures 2
			17b	For binary outcomes, presentation of both absolute and relative effect sizes is recommended	Not applicable
Ancillary analyses			18	Results of any other analyses performed, including subgroup analyses and adjusted analyses, distinguishing pre-specified from exploratory	6
Harms			19	All important harms or unintended effects in each group	6
Discussion					
Limitations			20	Trial limitations, addressing sources of potential bias, imprecision, and, if relevant, multiplicity of analyses	8
Generalisability			21	Generalisability (external validity, applicability) of the trial findings	7,8
Interpretation			22	Interpretation consistent with results, balancing benefits and harms, and considering other relevant evidence	7,8
Other information					
Registration			23	Registration number and name of trial registry	2
Protocol			24	Where the full trial protocol can be accessed, if available	Not applicable
Funding			25	Sources of funding and other support (such as supply of drugs), role of funders	13
